# Compositional action recognition with multi-view feature fusion

**DOI:** 10.1371/journal.pone.0266259

**Published:** 2022-04-14

**Authors:** Zhicheng Zhao, Yingan Liu, Lei Ma

**Affiliations:** College of Information Science and Technology, Nanjing Forestry University, Nanjing, China; PDPM IIITDM: PDPM Indian Institute of Information Technology Design and Manufacturing Jabalpur, INDIA

## Abstract

Most action recognition tasks now treat the activity as a single event in a video clip. Recently, the benefits of representing activities as a combination of verbs and nouns for action recognition have shown to be effective in improving action understanding, allowing us to capture such representations. However, there is still a lack of research on representational learning using cross-view or cross-modality information. To exploit the complementary information between multiple views, we propose a feature fusion framework, and our framework is divided into two steps: extraction of appearance features and fusion of multi-view features. We validate our approach on two action recognition datasets, IKEA ASM and LEMMA. We demonstrate that multi-view fusion can effectively generalize across appearances and identify previously unseen actions of interacting objects, surpassing current state-of-the-art methods. In particular, on the IKEA ASM dataset, the performance of the multi-view fusion approach improves 18.1% over the performance of the single-view approach on top-1.

## Introduction

**Fig 1:**
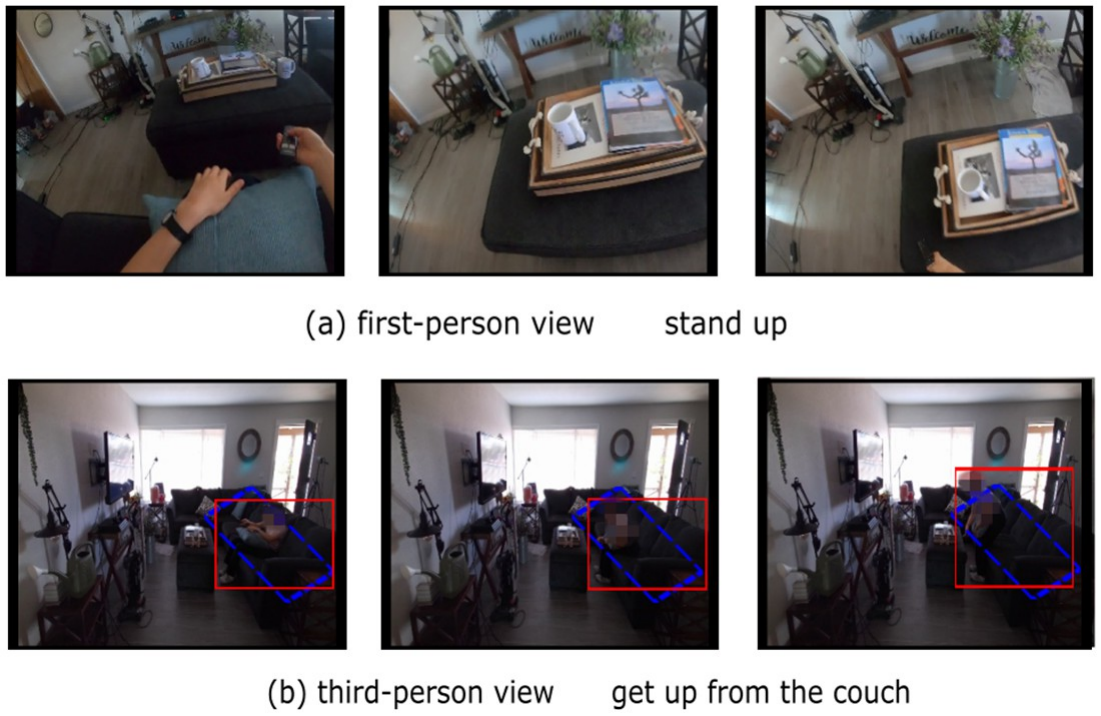
Examples of actions on LEMMA [3] dataset. (a) and (b) are images presented in different views at the same point in time. We have annotated the person by the red boxes and objects by the blue dashed boxes in (b). We can see the object moving away from the person and the person’s view is rising in (a), so we guess the action is standing up. But we can clearly see the person getting up from the couch in (b). We cannot recognize actions based on objects’ appearance in (a), but we can recognize actions in (b). Inspired by this, we aim at fusing information from different modalities for understanding compositional actions.

Recognizing human activity in natural scenes is a long-term challenge for deep learning approaches to activity recognition and is one of the most fundamental problems in artificial intelligence and computer vision. Humans can easily recognize actions by combining perceptual information about the behavior with knowledge about the socio-cultural, immediate context of the behavior and their own experience [1]. As shown in Fig 1(b), the activity is recognized by observing the change in relative position between two instances (i.e., person, couch). We expect that machines can have the similar capability by learning videos of human daily activities to facilitate future research on robotic tasks. This task of decomposing scenes into their corresponding subjects and objects and reasoning about the visual relationships between them is compositional action recognition. It is a classification task, similar to the image classification task [2].

Compositional action recognition studies the actions represented by the geometric changes between the subject (person) and the objects [4]. In the compositional action recognition setting, we decompose each action into combination of subjects, verbs and objects. In traditional setup [5], the training and testing sets have overlapping combination of verbs and nouns at the time of segmentation, but in our setup, we train and test our model on the same set of verbs, but force the nouns associated with the actions to be different, so that the combinations of actions and objects acting on the actions tested are not present at the time of training. Compositional action recognition only requires that the model is insensitive to the objects involved in the action and the appearance part of the scene where the action occurs. It is desirable that this task also extract features from other inputs (e.g., different views of the picture to deal with bodies, objects and self-obscuration), rather than just from a single view of the image. Obviously, positional features do not work for the same objects in different views, e.g., getting up from the couch in Fig 1(a). Therefore, fusing multi-view or multimodal features becomes a major problem for compositional action recognition.

In most cases, researchers use single-view analysis for action recognition [6]. Many powerful neural network structures [7, 8] have been developed for extracting visual representations from videos. These useful representations can achieve good performance in some simple action classification datasets, such as Breakfast [9] and Charades [10]. Some architectures contribute to action recognition tasks on other datasets or downstream video tasks after pre-training in large-scale datasets [11, 12]. To face more complex situations of action understanding, researchers started to use multi-view camera networks and multiple sensors for action capture, an approach that allows observing actions from different views and modalities. The traits of multi-view learning can improve the quality of visual representations [13, 14] because different views provide complementary information that can be used to help the learning of other views.

In this work, we propose a multi-view feature fusion framework that aims to improve visual representations. We argue that each single view sees only its specific pattern, and allowing views to share their unique information facilitates an improved overall perspective representation of the action. Each view sees different details and aspects of the action as it occurs, which is instructive for the other views to learn. At the same time, we process and reorganize the data from both datasets, dividing them according to the labels of the actions, so that they fit our compositional action setting.

We validate the proposed model on two action datasets, including IKEA ASM Dataset [15] and LEMMA [3], we first restructured the datasets according to the compositional setup, divided the training and testing sets, and then completed the compositional action recognition study on this basis. The experimental results show that the performance of our method is better than the state-of-the-art method [16–18] on the compositional action recognition task. Compared with [18], our model achieves an accuracy improvement of 2.4% and 6.2% for top-1 and top-3, respectively.

This paper is organized as follows: Section 1 is the introduction, the Section 2 is related work, the Section 3 is our methodology for this experiment, we introduced the experiment and visualization of some predictions in Sections 4 and 5. The last part is the conclusion.

## Related work

### Action recognition

In the last decade, action recognition can be divided into two phases: feature engineering and architecture engineering. In the first phase, STIP [19], Cuboids [20], dense trajectory [21], etc. were proposed, which take hand-crafted descriptors to be designed as spatio-temporal representations, these features perform well in some simple or controlled environments, but in the face of complex environments, the performance drops dramatically and lacks generality. In the second phase, many powerful neural architectures were developed to address the task of action recognition. Two-stream networks [22–24] were introduced, using two networks to model appearance and dynamics separately, and fusing the two streams by intermediate or final fusion. 3D CNNs [25–27] are effective in spatio-temporal feature extraction for video, extending common 2D CNNs with an additional temporal dimension. To improve the computational speed of the networks and reduce the training time, some architectures [28–31] balancing accuracy and computational efficiency have been designed. ACTION-Net [32] utilizes multipath excitation to obtain motion features, channel-wise features and spatio-temporal features for video action recognition. TDN [33] devises an efficient temporal module to capture multiscale temporal information for efficient action recognition by explicitly leveraging a temporal difference operator. However, most of these works are unimodal, and to fully express the action, we propose a learning framework based on multiple views of the video.

### Compositional activity recognition

Most current action recognition methods [34–37] focus on extracting features from the whole scene. To evaluate whether video models focus more on temporal inference or the appearance of frames, researchers have proposed more fine-grained benchmarks and structured tasks, including compositional activity recognition. Recent works have proposed some datasets for action recognition. For example, the Something-Else task [38], which extends the Something-Something dataset [39] with new annotations and a new compositional split, proposes a spatiotemporal interaction network that explicitly models changes in the geometric configuration between agents and objects. AVA [40] localizes the actors of actions, lemma [3] explores the nature of complex human activities in a goal-directed, multi-agent, multi-task environment with realistic labeling of composed atomic responses and their associated tasks. Cater [41] is a synthetic video dataset in which events are decomposed into spatial and several atomic actions in the temporal domain. The IKEA ASM dataset [15] is a multi-modal and multi-view video dataset of assembly tasks to enable rich analysis and understanding of human activities. Action Genome [42] decomposes actions into spatio-temporal scene graphs, which explain how objects and their relationships change when an action occurs. To understand a complex action, [4] projects positional, appearance, and semantic features across different spaces and promote the fusion process by an auxiliary prediction task. In this work, we concentrate on generalizing compositional actions to novel environments by interactively fusing the features of different views.

## Approach

### Problem statement

**Fig 2:**
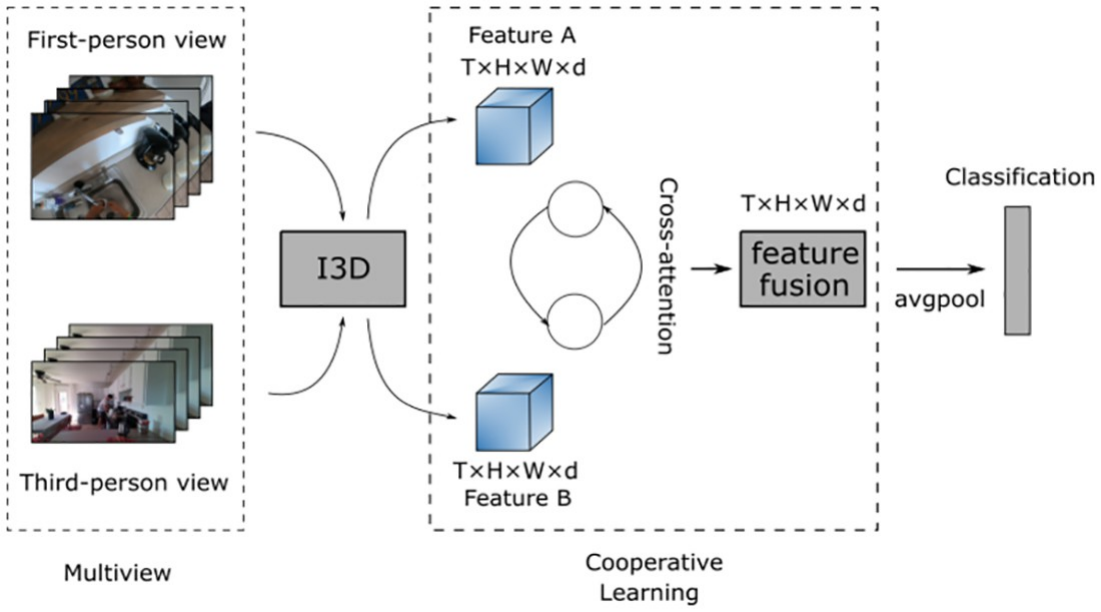
Overview of our model. Given the corresponding multiple views of RGB frames, we use I3D [43, 44] to compute features for each view, where different information about the action may exist for different views. These features are provided to cross attention as input and then classified after feature fusion.

Formally, given a video *V* with *T* frames, *H* × *W* resolution, *C* channels and *N* instances (e.g., objects and persons). We denote the RGB input of videos as {*I*_1_, *I*_2_, …, *I*_*T*_}, where $I\in R^{T\times H\times W\times C}$, and the action label of videos as {*l*_1_, *l*_2_, …, *l*_*T*_}, where *T* is the number of multimodalities. Fig 2 is overview of our model. Compositional action recognition aims at understanding the unseen combination of action (performed by subjects including persons) and objects in each video. And the purpose of multi-view human compositional action recognition is to assign queries to specific categories given a multi-view sample set of unknown actions captured from multiple views simultaneously.

As mentioned earlier, multiple views of features can be extracted from a video to understand complex activities. Multiple views exist for multimodal datasets, and for simplicity, we treat these multiple views as multiple independent modalities. For each modality there is a corresponding encoder to generate segment-level features. In simple terms, we expect one modality to provide some complementary information to other modalities during the training process. This is somewhat similar to existing approaches to knowledge distillation or student-teacher frameworks [45, 46]. However, we believe that this formulation of student/teacher is not suitable for a setup like ours, because we want the different modalities to be balanced with each other, rather than one of them being significantly more dominant than the others.

### Feature fusion framework

For this, we propose a simple and effective multi-view feature fusion framework that uses different view information to assist each other in extracting features and reasoning through compositional losses for action recognition. We first introduce the feature extraction part of the model, where we extract spatio-temporal features through a 3D ConvNet [43, 44] based on the ResNet-50 architecture. The input of our I3D model is in *T* × *H* × *W* × *C* dimensions, and the dimension of the filter kernel is denoted by *T* × *H* × *W*. The output dimension of the last convolution layer is *T* × *H* × *W* × *d* like the input dimension. Where d is the channel number. The backbone model of our approach, which are motivated by the network architecture introduced in [44].

#### Cooperative learning

**Fig 3:**
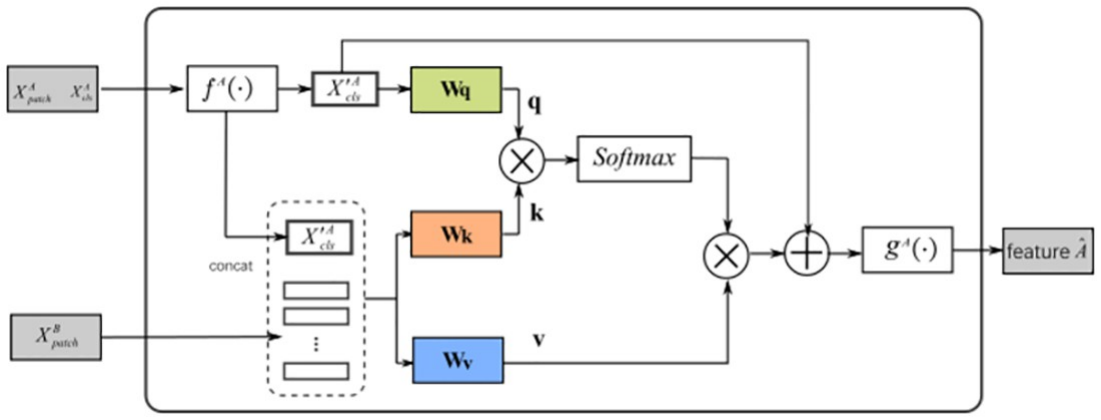
Cross-attention module for feature A branch. The CLS token of feature A serves as a query token to interact with the patch tokens from feature B through attention. *f*^*A*^(⋅) and *g*^*A*^(⋅) are linear projection functions for dimension alignment.

After extracting the features, we use Cross-attention module to fuse the features, after that, in feature fusion part, the corresponding elements of the two feature maps are added together and averaged to obtain a new *T* × *H* × *W* × *d* output features, which is our Cooperative learning. Fig 3 shows the Cross-attention module for feature A branch. In brief, for feature A, it first collects the patch tokens from the feature B and concatenates its own classification token (CLS) to them, as shown in Eq (1).

$$\begin{array}{c} X^{\prime A}=[f^{A}\left(X_{cls}^{A}\right)\left|\right|X_{patch}^{B}] \end{array}$$ (1)

where *f*^*A*^(⋅) is linear projection to align dimension. First, we observe that in self-attention the attention operation for each head is defined as the following.

$$\begin{array}{c} Attention\left(Q,K,V\right)=softmax\left(QK^{T}/\sqrt{d_{k}}\right)V \end{array}$$ (2)

the queries *Q* = *XW*_*q*_, keys *K* = *XW*_*k*_, and values *V* = *XW*_*v*_. They are linear projections of the input *X* with $X,Q,K,V\in R^{N\times d}$, *d*_*k*_ is keys of dimension. Cross-attention is not exactly the same as self-attention. Cross-attention (CA) is performed between $X_{cls}^{A}$ and *X*′^*A*^, where CLS token is a query and the information of patch tokens are integrated into CLS token. The CA can be mathematically expressed as follows.

$$\begin{array}{c} \begin{array}{c} q={X^{\prime }}_{cls}^{A}W_{q},k=X^{\prime A}W_{k},v=X^{\prime A}W_{v},\\ D=softmax\left(qk^{T}/\sqrt{C/h}\right),CA\left(X^{\prime A}\right)=Dv \end{array} \end{array}$$ (3)

where $W_{q},W_{k},W_{v}\in R^{C\times (C/h)}$ are the same as in self-attention [47], which are learnable parameters, C is the embedding dimension and h represents the number of heads. D is attention score. Softmax normalizes the relevancy value to [0, 1] and ensures that the sum of the probabilities of the individual predicted outputs is equal to 1. We conducted ablation study to find the best activation function of cross-attention module in Section 4. We only use CLS in the query, the generation of attention map (D) in cross-attention are linear. We use multiple heads in the CA(MCA). Layer normalization (LN) is applied before every block, and residual shortcuts after every block. The output $\hat{A}$ of a cross-attention module is defined as follows.

$$\begin{array}{c} \begin{array}{cc} y_{cls}^{A} & =f^{A}\left(X_{cls}^{A}\right)+MCA\left(LN\left([f^{A}\left(X_{cls}^{A}\right)\left|\right|X_{patch}^{B}]\right)\right)\\ \hat{A} & =[g^{A}\left(y_{cls}^{A}\right)\left|\right|X_{patch}^{A}] \end{array} \end{array}$$ (4)

where *f*^*A*^(⋅) and *g*^*A*^(⋅) are the projection functions for dimension alignment.

In addition to the multi-view nature of the datasets, they also have video-level action labels. These labels are useful for determining compositional actions as well as for learning action features. We measure the compositionality of activities and atomic actions in the model by utilizing both the labels of compositional actions and activity labels in the learning task. An intuitive understanding of our model is its ability to learn relationships between actions and compositional actions to improve its comprehension. We use the fused features to predict the category labels of videos and activities. The video action recognition task is a standard classification task, and we denote its corresponding loss as the loss of A branch: *L*_*a*_, and the loss of B branch: *L*_*b*_. The overall compositional loss is denoted by *L*_*compositional*_ = *L*_*c*_ = *L*_*a*_+ *L*_*b*_.

### Implementation details

We present the experimental details in the following three aspects.

#### Input

Our approach treats each view in the multi-view dataset as a modality, while taking as input T-frame information uniformly sampled from the video of each view, we resize the resolution of each input frame to 224 x 224.

#### Network architecture

In the task of action recognition, the frame-based feature extractor is an important part of it. We use the 3D convolutional networks built on ResNet-50 as the backbone of our model to extract spatio-temporal representations. To fuse the multi-view feature more effectively, we use the cross-attention model. Specifically, we first use the CLS token of each branch as a proxy to exchange information between the patch tokens of other branches. After fusing the abstract information in its own branch with the different information of another branch, the CLS token interacts with its own patch token and passes the learned information to the patch token, thus achieving the effect of fusing features. We use a dropout [48] with p = 0.3 on the last average pooling layer, and compute the final classification through the fully connected layer, the loss function is a simple cross entropy loss.

#### Training details

We train our model for 50 epochs using SGD optimizer, the momentum is 0.9 and the weight decay is 0.0001. The initial learning rate is 0.01, at epochs 15, 25, and 35, the learning rate decayed by a factor of 10.

## Experiment

We conducted experiments on the two proposed tasks: action recognition and compositional action recognition.

### Data processing

#### Dataset

**Fig 4:**
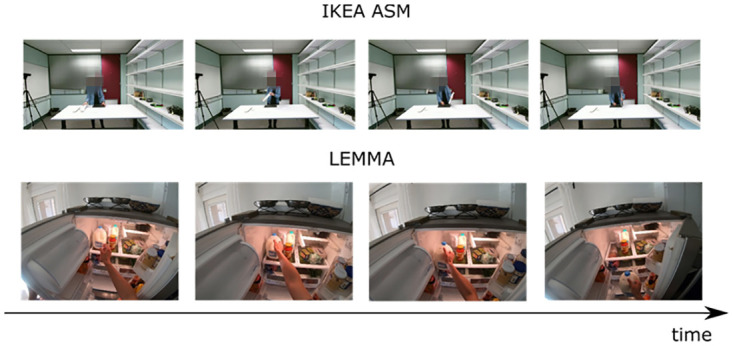
Action segments in the two datasets.

We selected two datasets to evaluate the proposed approach: i), the IKEA ASM dataset—a multi-modal and multi-view video dataset of assembly tasks. This is a novel furniture assembly dataset that includes multi-modal and multi-view annotated data, enabling rich analysis and understanding of human activities. The dataset has 16,764 actions with annotations; ii), the LEMMA dataset—a Multi-view Dataset for LEarning Multi-agent Multi-task Activities, containing 24 verb classes and 862 composed atomic-action tags. LEMMA dataset aims to explore the essence of complex human activities in a multi-task, multi-agent, goal-directed setting with ground-truth labels of compositional atomicactions and their associated tasks. The dataset has 800,000 frames with annotations. Fig 4 shows the action segments in the two datasets.

Human complex activities are composed of many action segments, and human activities are highly purposeful. The simplest verb of “take”, can generate a variety of different combinations of actions, such as “take the cup from the sink” or “take the book from the table”, which can generate a variety of combinations of verbs and nouns.

**Fig 5:**
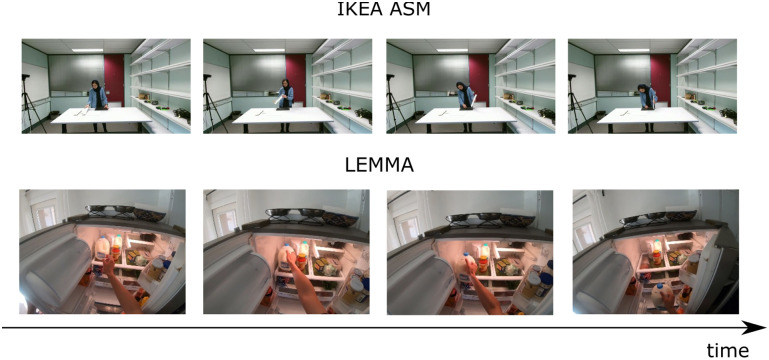
A small sample of compositional action. Red denotes verbs, blue indicates nouns for the interacting objects, yellow denotes location and green denotes tools to be used. If a compositional action consisting of a verb appears in the training set, then that compositional action cannot appear in the testing set. In a word, combinations of actions and objects are forced not to overlap between the training and testing sets.

Inspired by the above phenomena, we propose our setup for compositional action recognition, where we decompose each complex action into a combination of a verb and one or more nouns. These nouns can be an interactive object of the action, a location, or a tool to be used. A small sample of our compositional actions and how we divided the training and testing sets are shown in Fig 5. Unlike traditional training and test segmentation that includes the same verb and noun combination setup, we combine verbs with different nouns during training so that some new combination of verbs and objects emerges and is not seen by the model, and we require the model to be able to recognize the action. We reorganized the dataset and our goal is to enable model training to generalize to previously unseen action combinations, thus, we propose an action-based training/test split, where actions in the test environment do not appear in the training set and vice versa.

### Results on LEMMA dataset

Each video clip in this dataset has multiple action labels, of which there are 863 compositional action categories and 25 verb categories. So we remove the original prediction branch and use a binary cross-entropy loss and sigmoid layer to provide supervision to compositional action recognition for the multi-label classification problem. We reclassify LEMMA according to the compositional action setting. Specifically, suppose there are two different actions cut, eat, and two different nouns apple, bread. The purpose of the compositional action recognition is to be able to recognize some actions that have not been seen before. So we use combinations like cut apple, eat bread during training but cut bread, eat apple during testing, so that the different combinations of verbs and nouns are divided into training and testing parts. LEMMA is divided according to this setup and our statistical results are reported in Table 1. We take 32 frames (T = 32) from each video clip as input, and we train each model with a batch size of 8 on this dataset. We evaluate the performance of the model using the average precision, average recall and average F1-score predicted on the testing set.

**Table 1:** Statistics of original action recognition and compositional action recognition on LEMMA.

Task	# Classes	Training	Validation
Original	24	3450000	1150000
Compositional	24	52848	32929

We conducted experiments on this dataset for verb recognition and compositional action recognition, the results of the experiment are reported in Table 2. We can see that the average precision of verb recognition in FPV is 17.09%, the average precision of verb recognition in TPV is 14.18%, the average precision of compositional action recognition in FPV is 11.07%, the average precision of compositional action recognition in TPV is 6.85%, the results in FPV are significantly higher than those in TPV due to the difficulty of capturing action details in the third-person view, which indicates that our current method cannot obtain enough information about valid actions from TPV. The results of verb recognition are better than those of compositional action recognition, the average precision of verb recognition in combined view is 17.93%, the average precision of compositional action recognition in combined view is 11.82%, so the results of the combined view method are better than those of both TPV and FPV, indicating that the combined view is able to capture more action features.

**Table 2:** Compositional action recognition and verb recognition on LEMMA.

view	verb			compositional action		
	Avg.Prec	Avg.Rec	Avg.F1	Avg.Prec	Avg.Rec	Avg.F1
FPV	17.09	43.89	24.60	11.07	39.49	17.30
TPV	14.18	36.34	20.40	6.85	23.82	10.64
combined view	17.93	44.73	26.89	11.82	40.2	18.27

FPV indicates first-person view and TPV indicates third-person view.

#### Comparisons with the state-of-the-art method

We compare several state-of-the-art action recognition methods [16–18] that can be easily integrated into our framework, and for each method, we fine-tune the parameters provided in the original paper, and we also report the combination of different views by averaging the softmax output scores, as shown in the Table 3. From the results, we can know that the ResNet50 method has the worst performance, the average precision is only 6.93%. The average precisions of C3D and P3D methods are 8.26% and 10.34%, respectively. Our method has the best performance and the precision is 11.82%.

**Table 3:** Compositional action recognition on LEMMA.

method	Avg.Prec	Avg.Rec	Avg.F1
ResNet50 [16]	6.93	24.12	10.77
C3D [17]	8.26	31.23	13.06
P3D [18]	10.34	38.1	16.27
ours-I3D+CA	11.82	40.2	18.27

### Results on IKEA ASM dataset

All our models have 8 frames(T = 8) as input and we train each model with a batch size of 32 on this dataset. We divide the training and validation sets on the IKEA ASM dataset according to the compositional action setting, i.e., we ensure that the action labels in the training and validation sets do not overlap. The statistics and Comparison of the training and the validation sets are shown in Table 4. We use top-1, top-3, macro-recall by separately computing recall for each category and then averaging and mean average precision (mAP) those main metrics for evaluation.

**Table 4:** Statistics of original action recognition and compositional action recognition on IKEA ASM.

Task	# Classes	Training	Validation
Original	32	12812	5491
Compositional	6	9234	2151

#### Ablation study

We first performed some ablation studies to evaluate the performance of each component of our method in single view as well as in combined view. The results are summarized in Table 5.

**Table 5:** Ablation study of action recognition on IKEA ASM dataset.

method	view	Accuracy(%)		macro(%)	mAP(%)
		top-1	top-3		
I3D [15]	top view	57.6	76.6	39.2	28.4
	front view	60.8	79.3	39.6	29.1
	side view	52.2	72.2	38.7	27.5
	combined views	63.1	80.5	41.3	31.4
ours-I3D+CA	top view	76.3	94.8	56.4	43.8
	front view	77.5	95.1	56.7	44.3
	side view	71	92.9	55.7	43.1
	combined views	81.2	96.8	60.8	47.9

From the results, we can clearly see that the best single view method is front view, and the performance of the combined view method is improved compared to any of the single view methods. The accuracy of our feature fusion framework, i.e. ours-I3D+CA, is improved by 18.1% and 16.3% for top-1 and top-3 respectively on the combined view approach compared to I3D. The results also suggest that further research on methods for fusing and integrating the use of multi-view or multimodal data, driven by multi-view and multimodal datasets, should be conducted in future work.

We also conducted ablation study to compare the activation function of cross-attention module as discussed in Section 3. We conducted experiments on cross-attention module without and with different activation functions. The result is shown in Table 6. The first finding is that having an activation function is better than not having one, because without an activation function, the inner product is not guaranteed to be non-negative. Another finding is that Softmax is better than Sigmoid and Relu. Because the properties of softmax function can highlight the weights of important elements. Therefore, we use this setting throughout our experiments.

**Table 6:** Comparison of Softmax with other activation functions.

function	top-1(%)	top-3(%)	macro(%)	mAP(%)
none	27.3	53.8	20.5	10.4
Softmax	56.2	87.3	42.1	31.2
Sigmoid	43.7	68.7	31.2	20.6
Relu	49.3	76.1	34.3	23.9

#### Compositional action recognition

We further evaluated our model on a setup based on compositional action recognition. We report experiment performance on different views in Table 7. In the case that the training set data and the test set data do not contain or overlap each other, the front view method has the highest accuracy of 55.9% on top-1, while macro-recall is 39.7% and mAP is 29.4%. The side view method has the highest accuracy of 85.9% on top-3, macro-recall is 37.7%, mAP is 27.3%. The accuracy of the combined view method is 56.2% on top-1 and 87.3% on top-3, respectively, both of which are better than the performance of single view method. It indicates that the combined, holistic approach with multiple views or multimodal data in the setting of compositional action recognition helps to improve the performance of the model.

**Table 7:** Compositional action recognition of different views based on IKEA ASM dataset.

view	Accuracy(%)		macro(%)	mAP(%)
	top-1	top-3		
top view	55.6	79.4	38.3	27.6
front view	55.9	84.2	39.7	29.4
side view	52.2	85.9	37.7	27.3
combined view	56.2	87.3	42.1	31.2

The method used is ours-I3D+CA.

#### Comparisons with the state-of-the-art method

In line with the methods used in the comparison with LEMMA above, we also used these three methods for comparison on IKEA ASM dataset, and the results are presented in Table 8. The ResNet50 method has an accuracy of 29.4% on top-1, 55.1% on top-3, 22.1% macro-recall and 11.9% mAP. It has the worst performance. The performance of P3D method is better compared to C3D method and ResNet50 method. The P3D method has an accuracy of 53.8% on top-1, 81.1% on top-3, 39.6% macro-recall and 29.1% mAP. Overall, ours-I3D+CA outperforms all other methods, and we achieve 2.4% and 6.2% higher accuracy in top-1 and top-3, respectively, compared to [18], benefiting from the fusion of features facilitated by the cooperative learning task.

**Table 8:** Compositional action recognition on IKEA ASM dataset.

method	Accuracy(%)		macro(%)	mAP(%)
	top-1	top-3		
ResNet50 [16]	29.4	55.1	22.1	11.9
C3D [17]	44.9	68.6	32.3	21.8
P3D [18]	53.8	81.1	39.6	29.1
ours-I3D+CA	56.2	87.3	42.1	31.2

## Visualization

**Fig 6:**
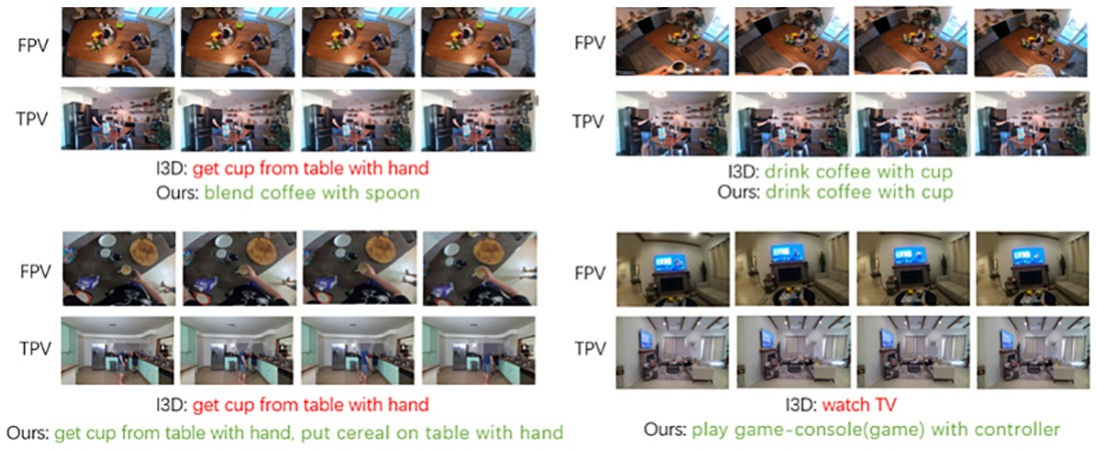
Predictions of I3D [3], and ours (ours-I3D + CA) on some examples from LEMMA dataset. Red means wrong prediction, green means correct prediction.

Fig 6 visualizes some predictions on LEMMA dataset. For clarity, only a few action classes are displayed. In the single-view case, I3D confuses when the action is similar to other action classes, while our model performs better in the multi-view case. And when the video clip contains multiple actions, I3D is not able to detect all the actions accurately in the single-view case, while our model usually performs better than the I3D model in the multi-view case.

## Conclusion

In this paper, we first point out the feasibility of using multi-view feature fusion methods on multi-view action recognition datasets. To address this issue, we propose a new multi-view fusion framework that enriches the video representation by exploiting the complementary information from different views. Experimental results on two action recognition datasets demonstrate the effectiveness of our multi-view learning framework in the compositional action recognition task. This work indicates that activity understanding is actually a process of fusing features from multiple sources, facilitating people to use richer feature representations in action recognition in the future. We hope that this effort will inspire future research on compositional activity understanding in real-world scenarios.

## References

[1] Keestra M. Understanding human action: integrating meanings, mechanisms, causes, and contexts. case studies in interdisciplinary research. 2012; p. 225–258. doi: 10.4135/9781483349541.n8

[2] Kaur A, Chauhan APS, Aggarwal AK. An automated slice sorting technique for multi-slice computed tomography liver cancer images using convolutional network. Expert Systems with Applications. 2021;186:115686. doi: 10.1016/j.eswa.2021.115686

[3] Jia B, Chen Y, Huang S, Zhu Y, Zhu Sc. Lemma: A multi-view dataset for learning multi-agent multi-task activities. In: European Conference on Computer Vision. Springer; 2020. p. 767–786.

[4] Yan R, Xie L, Shu X, Tang J. Interactive Fusion of Multi-level Features for Compositional Activity Recognition. arXiv preprint arXiv:201205689. 2020;.

[5] Ye Q, Huang P, Zhang Z, Zheng Y, Fu L, Yang W. Multiview Learning With Robust Double-Sided Twin SVM. IEEE Transactions on Cybernetics. 2021;. 10.1109/TCYB.2021.3088519 34546934

[6] Tang J, Shu X, Yan R, Zhang L. Coherence constrained graph LSTM for group activity recognition. IEEE transactions on pattern analysis and machine intelligence. 2019;. 10.1109/TPAMI.2019.2928540 31329548

[7] Lin J, Gan C, Han S. Tsm: Temporal shift module for efficient video understanding. In: Proceedings of the IEEE/CVF International Conference on Computer Vision; 2019. p. 7083–7093.

[8] Yan R, Xie L, Tang J, Shu X, Tian Q. Social adaptive module for weakly-supervised group activity recognition. In: European Conference on Computer Vision. Springer; 2020. p. 208–224.

[9] Kuehne H, Arslan A, Serre T. The language of actions: Recovering the syntax and semantics of goal-directed human activities. In: Proceedings of the IEEE conference on computer vision and pattern recognition; 2014. p. 780–787.

[10] Sigurdsson GA, Varol G, Wang X, Farhadi A, Laptev I, Gupta A. Hollywood in homes: Crowdsourcing data collection for activity understanding. In: European Conference on Computer Vision. Springer; 2016. p. 510–526.

[11] Carreira J, Noland E, Hillier C, Zisserman A. A short note on the kinetics-700 human action dataset. arXiv preprint arXiv:190706987. 2019;.

[12] Caba Heilbron F, Escorcia V, Ghanem B, Carlos Niebles J. Activitynet: A large-scale video benchmark for human activity understanding. In: Proceedings of the ieee conference on computer vision and pattern recognition; 2015. p. 961–970.

[13] Sayed N, Brattoli B, Ommer B. Cross and learn: Cross-modal self-supervision. In: German Conference on Pattern Recognition. Springer; 2018. p. 228–243.

[14] Tian Y, Krishnan D, Isola P. Contrastive multiview coding. In: Computer Vision–ECCV 2020: 16th European Conference, Glasgow, UK, August 23–28, 2020, Proceedings, Part XI 16. Springer; 2020. p. 776–794.

[15] Ben-Shabat Y, Yu X, Saleh F, Campbell D, Rodriguez-Opazo C, Li H, et al. The ikea asm dataset: Understanding people assembling furniture through actions, objects and pose. In: Proceedings of the IEEE/CVF Winter Conference on Applications of Computer Vision; 2021. p. 847–859.

[16] He K, Zhang X, Ren S, Sun J. Deep residual learning for image recognition. In: Proceedings of the IEEE conference on computer vision and pattern recognition; 2016. p. 770–778.

[17] Tran D, Bourdev L, Fergus R, Torresani L, Paluri M. Learning spatiotemporal features with 3d convolutional networks. In: Proceedings of the IEEE international conference on computer vision; 2015. p. 4489–4497.

[18] Qiu Z, Yao T, Mei T. Learning spatio-temporal representation with pseudo-3d residual networks. In: proceedings of the IEEE International Conference on Computer Vision; 2017. p. 5533–5541.

[19] Laptev I, Marszalek M, Schmid C, Rozenfeld B. Learning realistic human actions from movies. In: 2008 IEEE Conference on Computer Vision and Pattern Recognition. IEEE; 2008. p. 1–8.

[20] Dollár P, Rabaud V, Cottrell G, Belongie S. Behavior recognition via sparse spatio-temporal features. In: 2005 IEEE International Workshop on Visual Surveillance and Performance Evaluation of Tracking and Surveillance. IEEE; 2005. p. 65–72.

[21] Wang H, Kläser A, Schmid C, Liu CL. Dense trajectories and motion boundary descriptors for action recognition. International journal of computer vision. 2013;103(1):60–79. doi: 10.1007/s11263-012-0594-8

[22] Wang L, Xiong Y, Wang Z, Qiao Y, Lin D, Tang X, et al. Temporal segment networks: Towards good practices for deep action recognition. In: European conference on computer vision. Springer; 2016. p. 20–36.

[23] Simonyan K, Zisserman A. Two-stream convolutional networks for action recognition in videos. arXiv preprint arXiv:14062199. 2014;.

[24] Feichtenhofer C, Pinz A, Zisserman A. Convolutional two-stream network fusion for video action recognition. In: Proceedings of the IEEE conference on computer vision and pattern recognition; 2016. p. 1933–1941.

[25] Ji S, Xu W, Yang M, Yu K. 3D convolutional neural networks for human action recognition. IEEE transactions on pattern analysis and machine intelligence. 2012;35(1):221–231. doi: 10.1109/TPAMI.2012.59 22392705

[26] Feichtenhofer C, Fan H, Malik J, He K. Slowfast networks for video recognition. In: Proceedings of the IEEE/CVF international conference on computer vision; 2019. p. 6202–6211.

[27] Stroud J, Ross D, Sun C, Deng J, Sukthankar R. D3d: Distilled 3d networks for video action recognition. In: Proceedings of the IEEE/CVF Winter Conference on Applications of Computer Vision; 2020. p. 625–634.

[28] Tran D, Wang H, Torresani L, Ray J, LeCun Y, Paluri M. A closer look at spatiotemporal convolutions for action recognition. In: Proceedings of the IEEE conference on Computer Vision and Pattern Recognition; 2018. p. 6450–6459.

[29] Yan R, Xie L, Tang J, Shu X, Tian Q. HiGCIN: hierarchical graph-based cross inference network for group activity recognition. IEEE Transactions on Pattern Analysis and Machine Intelligence. 2020;. 10.1109/TPAMI.2020.3034233 33108281

[30] Zhou Y, Sun X, Zha ZJ, Zeng W. Mict: Mixed 3d/2d convolutional tube for human action recognition. In: Proceedings of the IEEE conference on computer vision and pattern recognition; 2018. p. 449–458.

[31] Xie S, Sun C, Huang J, Tu Z, Murphy K. Rethinking spatiotemporal feature learning: Speed-accuracy trade-offs in video classification. In: Proceedings of the European conference on computer vision (ECCV); 2018. p. 305–321.

[32] Wang Z, She Q, Smolic A. Action-net: Multipath excitation for action recognition. In: Proceedings of the IEEE/CVF Conference on Computer Vision and Pattern Recognition; 2021. p. 13214–13223.

[33] Wang L, Tong Z, Ji B, Wu G. Tdn: Temporal difference networks for efficient action recognition. In: Proceedings of the IEEE/CVF Conference on Computer Vision and Pattern Recognition; 2021. p. 1895–1904.

[34] Diba A, Fayyaz M, Sharma V, Arzani MM, Yousefzadeh R, Gall J, et al. Spatio-temporal channel correlation networks for action classification. In: Proceedings of the European Conference on Computer Vision (ECCV); 2018. p. 284–299.

[35] Ye Q, Li Z, Fu L, Zhang Z, Yang W, Yang G. Nonpeaked discriminant analysis for data representation. IEEE transactions on neural networks and learning systems. 2019;30(12):3818–3832. doi: 10.1109/TNNLS.2019.2944869 31725389

[36] Fu L, Li Z, Ye Q, Yin H, Liu Q, Chen X, et al. Learning robust discriminant subspace based on joint L2, p-and L2, s-Norm distance metrics. IEEE transactions on neural networks and learning systems. 2020;. 10.1109/TNNLS.2020.3027588 33180734

[37] Yan R, Tang J, Shu X, Li Z, Tian Q. Participation-contributed temporal dynamic model for group activity recognition. In: Proceedings of the 26th ACM international conference on Multimedia; 2018. p. 1292–1300.

[38] Materzynska J, Xiao T, Herzig R, Xu H, Wang X, Darrell T. Something-else: Compositional action recognition with spatial-temporal interaction networks. In: Proceedings of the IEEE/CVF Conference on Computer Vision and Pattern Recognition; 2020. p. 1049–1059.

[39] Goyal R, Ebrahimi Kahou S, Michalski V, Materzynska J, Westphal S, Kim H, et al. The” something something” video database for learning and evaluating visual common sense. In: Proceedings of the IEEE international conference on computer vision; 2017. p. 5842–5850.

[40] Gu C, Sun C, Ross DA, Vondrick C, Pantofaru C, Li Y, et al. Ava: A video dataset of spatio-temporally localized atomic visual actions. In: Proceedings of the IEEE Conference on Computer Vision and Pattern Recognition; 2018. p. 6047–6056.

[41] Girdhar R, Ramanan D. CATER: A diagnostic dataset for Compositional Actions and TEmporal Reasoning. arXiv preprint arXiv:191004744. 2019;.

[42] Ji J, Krishna R, Fei-Fei L, Niebles JC. Action genome: Actions as compositions of spatio-temporal scene graphs. In: Proceedings of the IEEE/CVF Conference on Computer Vision and Pattern Recognition; 2020. p. 10236–10247.

[43] Carreira J, Zisserman A. Quo vadis, action recognition? a new model and the kinetics dataset. In: proceedings of the IEEE Conference on Computer Vision and Pattern Recognition; 2017. p. 6299–6308.

[44] Wang X, Girshick R, Gupta A, He K. Non-local neural networks. In: Proceedings of the IEEE conference on computer vision and pattern recognition; 2018. p. 7794–7803.

[45] Kong Q, Wu Z, Deng Z, Klinkigt M, Tong B, Murakami T. Mmact: A large-scale dataset for cross modal human action understanding. In: Proceedings of the IEEE/CVF International Conference on Computer Vision; 2019. p. 8658–8667.

[46] Hinton G, Vinyals O, Dean J. Distilling the knowledge in a neural network. arXiv preprint arXiv:150302531. 2015;.

[47] Vaswani A, Shazeer N, Parmar N, Uszkoreit J, Jones L, Gomez AN, et al. Attention is all you need. In: Advances in neural information processing systems; 2017. p. 5998–6008.

[48] Hinton GE, Srivastava N, Krizhevsky A, Sutskever I, Salakhutdinov RR. Improving neural networks by preventing co-adaptation of feature detectors. arXiv preprint arXiv:12070580. 2012;.

